# Ivermectin Treatment for Strongyloidiasis in Thailand

**DOI:** 10.4269/ajtmh.24-0259

**Published:** 2024-07-02

**Authors:** Akira Hokama

**Affiliations:** Department of Medical Checkup Naha City Hospital Naha, Okinawa, Japan E-mail: hokamaakira@gmail.com

Dear Sir,

I read with interest the article “High Efficacy of Ivermectin for Strongyloidiasis Treatment,” which described how a single dose of ivermectin (200 μg/kg) successfully eliminated *Strongyloides stercoralis* infection in asymptomatic individuals in northeast Thailand, with a 100% cure rate.^1^ I congratulate the authors on their efforts and fully agree with their highlighting ivermectin as a primary treatment of strongyloidiasis in an endemic community. I would like to comment on the possible reasons why they have achieved a 100% cure rate. It is well known that co-infection with human T-cell lymphotropic virus type 1 (HTLV-1) has been associated with a high rate of strongyloidiasis treatment failure.^2^^,^^3^ Hyperinfection syndrome and disseminated strongyloidiasis frequently occur with HTLV-1 co-infection,^2^ resulting in a high mortality rate despite repeated administration of ivermectin. In a prior study treatment with ivermectin (200 μg/kg twice) for strongyloidiasis led to parasite clearance in 100% (42/42) of patients without HTLV-1 co-infection, whereas it was 90% (18/20) in those with the co-infection.^4^ A large scale serological survey affirmed that HTLV has not been endemic in Thailand.^5^ I therefore speculate, that although the virological backgrounds of the volunteers were not described, the non-endemic status of HTLV-1 contributed to the 100% cure rate in the new study. Co-infection with another immunocompromising factor, human immunodeficiency virus (HIV), has been a serious healthcare problem in Asia,^6^ and the efficacy of ivermectin against strongyloidiasis has not been evaluated for asymptomatic individuals with HIV infection. In conclusion, although the new study provides valuable information on ivermectin treatment for asymptomatic strongyloidiasis, future research, including consideration of virological backgrounds, is needed before concluding that single-dose ivermectin treatment is sufficient for asymptomatic strongyloidiasis in Thailand.
